# Effiziente Planung der Schlafendoskopie vor atmungsabhängiger unilateraler Zungenschrittmacherimplantation

**DOI:** 10.1007/s00106-025-01655-8

**Published:** 2025-08-28

**Authors:** Arne Böttcher, Lisa Schmitz, Linda J. Voß, Christian S. Betz, Jacob F. Clausen

**Affiliations:** 1 Klinik und Poliklinik für Hals‑, Nasen- und Ohrenheilkunde, Universitätsklinikum Hamburg-Eppendorf, Martinistraße 52, 20246 Hamburg, Deutschland; 2 Klinik für Audiologie und Phoniatrie, Charité – Universitätsmedizin Berlin, Berlin, Deutschland

**Keywords:** Obstruktive Schlafapnoe, Schlafbezogene Atmungsstörungen, Hypoglossusnervstimulation, Müller-Manöver, Komplett-konzentrischer Velopharynxkollaps, Obstructive sleep apnea, Sleep related breathing disorders, Hypoglossal nerve stimulation, Mueller’s maneuver, Complete concentric velopharyngeal collapse

## Abstract

**Einleitung:**

Die Implantation des atmungsabhängigen unilateralen Zungenschrittmachers erfordert bislang den präoperativen Ausschluss eines komplett konzentrischen Velopharynxkollaps (CCC) in der Schlafendoskopie („drug-induced sleep endoscopy“, DISE). Dies stellt das durchführende Implantationsteam vor gewisse logistische und betriebswirtschaftliche Herausforderungen. Um hier einen patientenfreundlichen sowie kosten- und zeiteffizienten Ablauf zu generieren, wurde das Eppendorfer DISE-Konzept entwickelt.

**Material und Methoden:**

Wir führten eine retrospektive Analyse von Patienten mit Zungenschrittmachern durch, die präoperativ mittels Müller-Manövers und simulierten Schnarchens im Liegen transnasal flexibelendoskopisch hinsichtlich eines CCC untersucht wurden. Bei unauffälligem Befund ohne Hinweise auf einen CCC wurde die DISE unmittelbar vor der Implantation des Zungenschrittmachers im selben Eingriff durchgeführt.

**Ergebnisse:**

Es wurden insgesamt *n* = 28 Patienten untersucht. Hierbei zeigte sich bei 7,9 % ein auffälliger Befund im Müller-Manöver bzw. beim simulierten Schnarchen. Diese Patienten zeigten in der DISE einen CCC. Bei unauffälligem Müller-Manöver zeigte sich auch in der DISE kein Anhalt für einen CCC. Somit lagen eine Sensitivität und eine Spezifität von jeweils 100 % vor. Gleiches gilt für den positiven bzw. negativen prädiktiven Wert. Somit kann eine direkte Abhängigkeit des Müller-Manövers vom velopharyngealen DISE-Befund gesehen werden (exakter Test nach Fisher: 0,0026; *p* < 0,05).

**Schlussfolgerung:**

Das Eppendorfer DISE-Konzept erweist sich als zuverlässige Methode, um einen CCC patientenorientiert sowie kosten- und zeiteffizient auszuschließen. Ob der CCC weiterhin ein Ausschlusskriterium für die unilaterale atmungsabhängige Zungenschrittmacherimplantation bleibt, bleibt abzuwarten.

## Abstract (en)

**Introduction:**

The implantation of breathing-dependent unilateral hypoglossal nerve stimulation (HNS) has traditionally required the preoperative exclusion of complete concentric velopharyngeal collapse (CCC) through drug-induced sleep endoscopy (DISE). This prerequisite poses logistical and operational challenges for implantation teams. To address these issues the Eppendorf DISE concept was developed to streamline the process, making it more patient-friendly, cost-effective, and time-efficient.

**Material and methods:**

We retrospectively examined HNS patients who were preoperatively examined for CCC using the Müller maneuver and simulated snoring in a supine position using transnasal flexible endoscopy. If the findings were unremarkable, with no evidence of CCC, the DISE was performed directly before the implantation in the same procedure.

**Results:**

We examined 28 patients of whom 7.9% presented remarkable findings during the Müller maneuver or simulated snoring. These patients also showed a CCC during DISE. If the Müller maneuver was unremarkable no CCC was observed in DISE either, resulting in 100% sensitivity, specificity, positive and negative predictive values. Thus, there is a significant direct relationship between Müller maneuver results and velopharyngeal findings during DISE as confirmed by Fisher’s exact test (*p* = 0.0026, *p* < 0.05).

**Conclusion:**

The Eppendorf DISE concept has shown to be a reliable method for predicting a low likelihood of CCC in a patient-oriented, cost-effective and time-efficient manner. It is yet to be determined how long the CCC will remain as an exclusion criterion for breathing-dependent unilateral HNS implantation.

## Einleitung

Die Implantation des unilateralen atmungsabhängigen Zungenschrittmachers („upper airway stimulation“, UAS) der Firma Inspire Medical Systems ist eine seit 2014 durch die Food And Drug Administration (FDA) zugelassene Alternativtherapie für Patienten mit mittel- bis schwergradiger obstruktiver Schlafapnoe (OSA) und PAP(„positive airway pressure“)-Nonadhärenz. Sie zeigt eine langfristig sehr hohe Effektivität bezüglich der Reduktion des Apnoe-Hypopnoe-Index (AHI), der Reduktion der Tagesschläfrigkeit und einer Erhöhung der schlafbezogenen Lebensqualität [1] und ist ein leitliniengerechter fester Bestandteil im operativen Repertoire vieler Kliniken [2, 3]. In einer Studie mit 21 Patienten konnte im Jahr 2013 gezeigt werden, dass Patienten mit einem komplett konzentrischen Velopharynxkollaps („complete concentric collapse“, CCC) in der Schlafendoskopie („drug-induced sleep endoscopy“, DISE) eine geringere AHI-Reduktion durch eine UAS aufwiesen [4]. Aus diesem Grund wurde der CCC als Ausschlusskriterium für dieses Implantat definiert und eine DISE vor UAS-Implantation erforderlich.

Als singulärer Eingriff im stationären Setting bedeutet dies für den Patienten einen zusätzlichen Eingriff vor der Implantation des Schrittmachers, zumeist unter Propofol-TCI („target-controlled infusion“). Für die Klinik ergibt sich in der Regel eine nicht kostendeckende Prozedur mit erheblichem Mehraufwand. Aktuell wird für stationäre Fälle (1 Übernachtung) die DRG („diagnosis related group“) E63B berechnet, was laut durchschnittlicher Kostenkalkulation einen Verlust von etwa 36 € pro gesetzlich versicherten Fall zur Folge hat. Bei einer Entlassung am selben Tag wäre der Deckungsbeitrag von etwa 86 € zwar positiv, könnte jedoch aufgrund der prinzipiellen ambulanten Durchführbarkeit vom medizinischen Dienst angefochten werden. Zudem führt die derzeitige Hochschulambulanzpauschale von 165 € zu einem Verlust von etwa 265 € und bietet somit keine Kostendeckung.

Es besteht demnach der Bedarf, das präoperative Prozedere effizienter und v. a. patientenfreundlicher zu gestalten. In der Literaturrecherche ergaben sich Hinweise darauf, dass ein sog. Müller-Manöver (nach Johannes Müller, dt. Physiologe, 1801–1858; [5]) wertvolle Hinweise zum pharyngealen Kollapsmuster bereits im präklinischen Setting liefern könnte. Bei diesem auch „negativer Valsalva-Versuch“ genannten Manöver wird während der transnasalen flexibel-endoskopischen Untersuchung der Patient gebeten, den Mund zu schließen, mit Zeigefinger und Daumen die Nasenweichteile mit einliegendem Endoskop zu verschließen und mehrere Inspirationsversuche durchzuführen [6, 7]. Bei korrekter Position des Endoskops mit vollständiger Einsicht des Nasopharynx kann das Velopharynxkollapsmuster abgeschätzt werden. Zur Erhöhung der klinischen Realitätsnähe wird dies im Liegen durchgeführt. Der Patient wird zudem anschließend gebeten, ein velares Schnarchen zu simulieren, um evtl. zusätzliche Kollapsmuster detektieren zu können [8].

## Material und Methoden

Wir analysierten retrospektiv alle zungenschrittmacherimplantierten Patienten mit präoperativ dokumentiertem Müller-Manöver und simuliertem Schnarchen (siehe oben). Hierbei wurde eine rein dichotome Negativanalyse auf Vorliegen bzw. Nichtvorliegen einer Pathologie (CCC) ohne weitere Graduierung bzw. Unterklassifikation des Kollapsverhaltens unter Müller-Manöver vorgenommen. Zusätzlich wurde in gleicher Methodik das simulierte Schnarchen hinzugezogen, um evtl. andere Kollapsmuster zu detektieren. Alle Befunde (Müller-Manöver und simuliertes Schnarchen wie auch DISE) wurden vom Erstautor der vorliegenden Studie abgenommen und final bewertet (teilweise im Nachgang per Videoaufzeichnung).

Der Einschluss erfolgte über das OP(Operation)-Managementprogramm Torin (Getinge) unter dem OPS(Operationen- und Prozedurenschlüssel)-Code 5‑095.c7 bzw. 1–611,01 und über das Krankenhausinformationssystem Soarian® Clinicals (Cerner).

Die DISE wurde in Propofol-TCI mit Entropiemessung via Bispektralindex (BIS) in Rückenlage durchgeführt. Bei ausreichender Sedierung (BIS ≈ 70) erfolgt das Einführen des Fiberendoskops durch eine Nasenhaupthöhle zunächst zur Nasopharyngoskopie. Die Evaluation des laryngopharyngealen Kollapsmusters erfolgte nach den VOTE(„velum/oropharynx/tongue base/epiglottis“)-Kriterien [9] bei einem BIS von weniger als 60. In unserer Klinik verzichten wir primär auf die Anwendung von abschwellenden Nasentropfen und Oberflächenanästhetika, da diese eine Mukosasekretionsinduktion hervorrufen können. Zudem wird standardmäßig kein Glycopyrroniumbromid (Robinul®) verabreicht, da es unter Berücksichtigung dieser Aspekte und der Nutzung der im Endoskop integrierten Absaugung nicht erforderlich ist.

Es erfolgte die statistische Berechnung mit Microsoft Excel 2021 sowie IBM SPSS Statistics (Version 29.0.1.0 [171]). Die Signifikanz auf Unabhängigkeit wurde mittels Fisher’s Exact Test berechnet. Die statistische Signifikanz wurde für *p* < 0,05 festgelegt.

## Ergebnisse

**Abb. 1:**
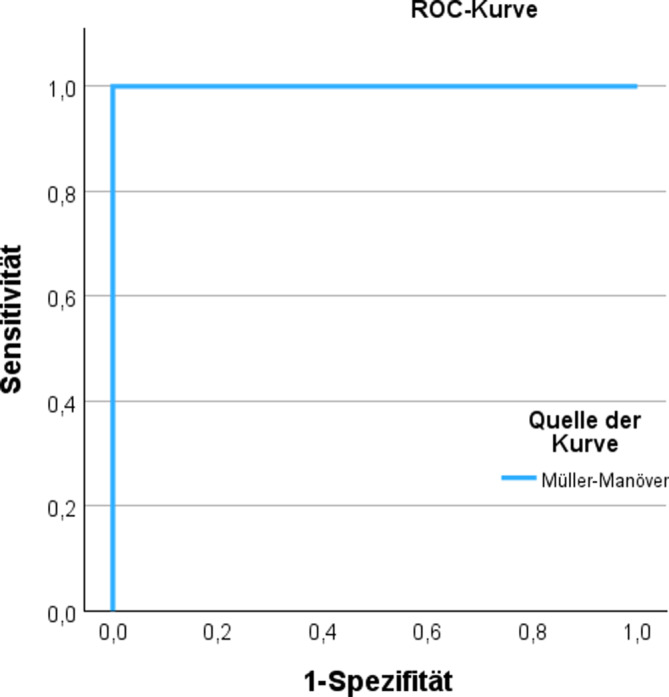
*ROC*(„receiver-operating characteristic“)-Kurve für die Modellgenauigkeit des Müller-Manövers im Liegen auf die Vorhersagbarkeit des komplett konzentrischen Velopharynxkollapses in der Schlafendoskopie

Wir identifizierten *n* = 28 Patienten (28,6 % weiblich/71,4 % männlich) mit dokumentiertem präoperativen Müller-Manöver und simuliertem Schnarchen vor Zungenschrittmacherimplantation. Die präoperativen klinischen Daten sind in Tab. 1 zusammengefasst. Es zeigten sich keine Unterschiede im velopharyngealen Kollapsverhalten zwischen Müller-Manöver und simuliertem Schnarchen. In 26 von 28 Fällen zeigte das Müller-Manöver ein unauffälliges Ergebnis ohne Hinweise auf einen CCC, was sich in allen 26 Fällen in der DISE bestätigte. In 2 Fällen wurde präoperativ ein auffälliges Kollapsmuster festgestellt, welches sich jeweils als CCC in der DISE erwies (Prävalenz: 7,9 %). Als auffällig wurden die Befunde klassifiziert, die inkonklusiv waren und bei denen es somit nicht zu einem eindeutigen „k. A. für CCC“ aus Sicht des Endoskopierers reichte. Wir sahen keine unterschiedlichen Kollapsmusterklassifikationen (dichotom – CCC vorliegend vs. CCC nicht vorliegend) zwischen Müller-Manöver und simuliertem Schnarchen (Tab. 2). Die stochastische Analyse ergab eine Sensitivität und Spezifität des Müller-Manövers von jeweils 100 %, verbunden mit einem positiven und negativen prädiktiven Wert von ebenfalls 100 % (Tab. 3). Dies spiegelt sich in der ROC(„receiver-operating characteristics“)-Kurve wider (Abb. 1). Im Fisher’s Exact Test zeigte sich für abhängige Variablen ein Wert von 0,0026 (*p* < 0,05).

**Tab. 1:** Patientendaten (*n* = 28)

Parameter	Mittelwert ± Standardabweichung	Spannweite (Min.–Max.)
Alter (Jahre)	59,6 ± 11,1	29–77
BMI	29,2 ± 3,7	21,8–34,6
AHI	32,4 ± 11,9	16,8–61,8
ODI	30,7 ± 17,4	9,8–76,8
ESS	12,7 ± 5,4	3–24

*AHI* Apnoe-Hypopnoe-Index, *BMI* Body-Mass-Index, *ESS* Epworth Sleepiness Scale,* ODI* „oxygen-desaturation index“

**Tab. 2:** Übersicht der erhobenen Befunde

Müller-Manöver (liegend)	Simuliertes Schnarchen (liegend)	Kombiniert	DISE – V				DISE – O		DISE – T		DISE – E			
[1 = V. a. komplett konzentrisch, 2 = k. A. komplett konzentrisch, 3 = inkonklusiv; 5 = n/a]	[1 = V. a. komplett konzentrisch, 2 = k. A. komplett konzentrisch, 3 = inkonklusiv; 5 = n/a]		Grad der Obstruktion [%]	A.-p. [%]	Lateral [%]	Konzentrisch [%]	Grad der Obstruktion [%]	Lateral [%]	Grad der Obstruktion [%]	A.-p. [%]	Grad der Obstruktion [%]	A.-p. [%]	Lateral [%]	Konzentrisch [%]
1/3	1/3	3	100	0	0	100	100	100	20	20	20	20	0	
1/3	1/3	3	100	0	0	100	100	100	70	70	100	20	80	
2	2	2	100	80	20	0	50	50	80	80	90	90	0	
2	2	2	100	100	0	0	100	100	50	50	50	20	30	
2	2	2	100	70	30	0	100	100	100	100	100	100	0	
2	2	2	100	90	10	0	60	60	20	20	20	20	0	
2	2	2	100	80	20	0	100	100	50	50	90	90	0	
2	2	2	100	90	10	0	20	20	100	100	100	100		
2	2	2	100	80	20	0	50	50	90	90	100	100	0	
2	2	2	100	75	25	0	75	75	75	75	75	75	0	
2	2	2	100	80	20	0	50	50	75	75	75	75	0	
2	2	2	100	75	25	0	50	50	50	50	100	100	0	
2	2	2	100	100	0	0	40	40	50	50	100	100	0	
2	2	2	100	50	50	0	100	100	*n. b.*	*–*	*n. b.*	–	–	
2	2	2	100	60	40	0	90	90	60	60	100	100	0	
2	2	2	100	50	50	0	80	80	60	60	80	0	80	
2	2	2	100	100	0	0	20	20	100	100	100	100	0	
2	2	2	100	70	30	0	100	100	100	100	100	100	0	
2	2	2	100	90	10	0	40	40	100	100	100	100	0	
2	2	2	100	100	0	0	50	50	30	30	50	50	0	
2	2	2	100	50	50	0	100	100	70	70	100	100	0	
2	2	2	100	70	30	0	30	30	50	50	100	100	0	
2	2	2	100	80	20	0	10	10	100	100	100	100	0	
2	2	2	100	60	40	0	100	100	30	30	100	100	0	
2	2	2	100	90	10	0	30	30	70	70	90	90	0	
2	2	2	100	80	20	0	30	30	100	100	100	100	0	
2	2	2	100	100	0	0	20	20	100	100	100	100	0	
2	2	2	100	70	30	0	50	50	80	80	100	100	0	

*DISE* „drug-induced sleep endoscopy“, *k.* *A.* kein Anhalt für, *n/a* not applicable, *n.* *b.* intraoperativ nicht befundbar

Anm.: Die prozentuale Graduierung des Kollapsverhaltens ist eine institutionsspezifische Modifikation der ursprünglichen VOTE(„velum/oropharynx/tongue base/epiglottis“)-Klassifikation.

**Tab. 3:** Kontingenztafel und Stochastik

	**DISE-Befund (Velopharynx)**		Spezifität	100 %
Müller-Manöver und simuliertes Schnarchen	CCC	Kein CCC	Sensitivität	100 %
Inkonklusiv (CCC nicht auszuschließen)	2	0	PPV	100 %
K. A. für CCC	0	26	NPV	100 %

*CCC* „complete concentric collapse“, *DISE* „drug-induced sleep endoscopy“, *PPV* „positive predictive value“, *NPV* „negative predictive value“

## Diskussion

Wir konnten eine sehr hohe Übereinstimmung zwischen dem velopharyngealen Kollapsmuster im Wachzustand, ermittelt durch das Müller-Manöver und simuliertes Schnarchen im Liegen, und den Ergebnissen während einer DISE unter Propofol-TCI nachweisen. Unsere Ergebnisse legen nahe, dass lediglich bei einem geringen Anteil an Patienten mit inkonklusivem Untersuchungsbefund im Wachzustand eine singuläre DISE im Vorfeld erforderlich wird. Zeigt sich im Müller-Manöver/simulierten Schnarchen kein Anhalt für einen CCC, kann eine DISE der Implantation im selben Eingriff direkt vorangestellt werden. Mithilfe unserer Ergebnisse ist ein deutlich erleichtertes, patientenschonenderes und effizienteres präoperatives Prozedere der UAS-Implantation möglich.

Die signifikante Korrelation des velopharyngealen Kollapsmusters während des Müller-Manövers im Liegen mit dem DISE-Befund bedeutet die Möglichkeit der Etablierung des Müller-Manövers als eine neue Standarduntersuchung für OSA-Patienten. Gleiches gilt für das simulierte Schnarchen. Ein wichtiger Aspekt hierbei ist jedoch die Befundung anhand hoher Qualitätsstandards, welche durch systematisches Training (z. B. durch Kurse, Hospitationen oder Online-Webinare) und Praxiserfahrung in der Schlafendoskopie erzielt werden können. Eine Befundung der DISE-Ergebnisse wurde zudem nur von schlafmedizinisch weitergebildetem fach- und oberärztlichen Personal (synchron oder metachron, also im Nachgang, per Videodokumentation) durchgeführt.

Die Veränderung des laryngopharyngealen Kollapsmusters durch unterschiedliche Sedierungstiefen ist weitgehend bekannt [10], scheint aber anhand unserer Daten im Bereich des Velopharynx für einen CCC keine klinisch relevante Rolle zu spielen. Eine standardisierte Propofol-TCI kann zusätzliche Schwankungen der Sedierungstiefe reduzieren [11].

Generell ist der CCC mit einer Prävalenz von hier 7,9 % bzw. 6,1 % aller in unserer SBAS(schlafbezogene Atmungsstörungen)-Sprechstunde schlafendoskopierten OSA-Patienten ([12]; bei Erfüllung aller weiteren Einschlusskriterien [AHI: 15–65, BMI: ≤ 35, Anteil gem./zentr. Apnoen: ≤ 25 % gemäß Weißbuch]; [13]) deutlich seltener als in früheren OSA-Kohorten angegeben (Spannweite: 17,9–37,3 %; [14–17]). Als Risikofaktoren wurden hierbei hohe Zungenposition (nach [14]), ausgeprägte Adipositas (BMI > 30; [18]), männliches Geschlecht [19], längere Schnarchdauer [15] und/oder sonstige anatomische Auffälligkeiten wie erhöhter Halsumfang (Männer: > 16 inch = 40,6 cm; [20]) oder ein Abstand zwischen den hinteren Gaumenbögen von weniger als 20 mm in Rückenlage identifiziert [21]. Nach Autorenmeinung ist eine Tonsillenhyperplasie für den CCC zumindest mitverantwortlich, wenngleich durch Studien noch nicht vollständig belegt [15, 19]. Eine Kausalität lässt sich vermuten, da nach erfolgter Tonsillektomie ± Uvulopalatopharyngoplastik bei 85,7–81,5 % der Patienten kein CCC in der DISE mehr nachweisbar ist [19, 22].

Die vorgestellten Daten unterliegen gewissen Limitationen. Das retrospektive Design und die nur mäßig standardisierbare DISE mit Evaluation nach VOTE-Kriterien bergen Potenzial für Fehlinterpretationen [23]. Bei ungeübter Befundung besteht die Gefahr, dass durch hyperplastische Seitenstränge, Rachenmandelpolster und Tubenwülste, z. B. durch den mit der OSA vergesellschafteten laryngopharyngealen Reflux [24], ein CCC vorgetäuscht werden kann. Die in Tab. 2 aufgeführte prozentuale Graduierung des Kollapsverhaltens während der DISE ist institutionsspezifisch und weicht somit von der klassischen VOTE-Einteilung (0: „no obstruction“ [„no vibration“], 1: „partial obstruction“ [„vibration“], 2: „complete obstruction“ [„collapse“], X: „not visualized“; [9]) ab. Hier bedarf es sicher einer weiteren Validierung.

Obwohl die Kohorte mit 28 Patienten relativ klein ist, zeigt sie eine bemerkenswerte Korrelation der Ergebnisse.

Das Müller-Manöver wurde in der Vergangenheit auch kritisch beurteilt, und es wurde sogar empfohlen, selbiges in aufrechter Position durchzuführen, da beim wachen Patienten in Rückenlage der M. tensor veli palatini aktiviert und somit ein verfälschtes Kollapsmuster abgeleitet werden würde [25]. Hierzu gibt es jedoch keine wissenschaftliche Evidenz [26, 27], und die Autoren sehen durch das Liegen eine realitätsnähere Aussagekraft der erhobenen Befunde.

Inwieweit der CCC weiterhin ein Ausschlusskriterium für eine UAS-Versorgung darstellen wird, bleibt abzuwarten. Es zeigen sich Hinweise, dass der komplette, laterale Tonsillen- bzw. Oropharynxkollaps als besser geeignetes Ausschlusskriterium fungieren könnte, um sog. Therapieversager vor einer UAS-Versorgung zu selektieren [28]. Eine Eignung des Müller-Manövers im Liegen sollte diesbezüglich genauer untersucht und die Kohorte entsprechend erweitert werden.

## Schlussfolgerung

Die Abschätzung eines CCC kann zuverlässig durch das Müller-Manöver und das simulierte Schnarchen im Liegen erfolgen. Dadurch kann die Anzahl singulärer DISE deutlich reduziert werden. Für einen Großteil der Patienten kann ein kombinierter Eingriff mit Zungenschrittmacherimplantation und vorangestellter DISE erfolgen. Durch das Eppendorfer DISE-Konzept ergeben sich sowohl für die Patienten als auch für die Kliniken große Vorteile.

## Data Availability

Die in dieser Studie erhobenen Datensätze können auf begründete Anfrage beim Korrespondenzautor angefordert werden.
